# Recent Advances in the Use of Iron–Gold Hybrid Nanoparticles for Biomedical Applications

**DOI:** 10.3390/nano11051227

**Published:** 2021-05-06

**Authors:** Mariam Abdulaziz M. Tarkistani, Varsha Komalla, Veysel Kayser

**Affiliations:** Sydney Pharmacy School, Faculty of Medicine and Health, The University of Sydney, Camperdown, NSW 2006, Australia; mtar8733@uni.sydney.edu.au (M.A.M.T.); varsha.komalla@sydney.edu.au (V.K.)

**Keywords:** nanomaterials, nanohybrids, magnetic plasmonic nanoparticles, gold nanoparticles, iron oxide nanoparticles, surface functionalization, biomedical applications

## Abstract

Recently, there has been an increased interest in iron–gold-based hybrid nanostructures, due to their combined outstanding optical and magnetic properties resulting from the usage of two separate metals. The synthesis of these nanoparticles involves thermal decomposition and modification of their surfaces using a variety of different methods, which are discussed in this review. In addition, different forms such as core–shell, dumbbell, flower, octahedral, star, rod, and Janus-shaped hybrids are discussed, and their unique properties are highlighted. Studies on combining optical response in the near-infrared window and magnetic properties of iron–gold-based hybrid nanoparticles as multifunctional nanoprobes for drug delivery, magnetic–photothermal heating as well as contrast agents during magnetic and optical imaging and magnetically-assisted optical biosensing to detect traces of targeted analytes inside the body has been reviewed.

## 1. Introduction

Nanoparticles (NPs) usually possess distinct chemical and physical properties compared to macromolecules, mainly due to their smaller sizes. These properties include conductance, magnetism, chemical interactions, and optical properties [1] as well as surface area, shape, magnetism, and permeability, affecting their characteristics and making them unique in their functioning [2].

NPs are ubiquitously present around us in nature, for instance, in our food, e.g., casein micelles [3]; in the biological matter such as viruses [4]; and other environmental sources such as ash, sand, and dust [5]. Due to their smaller sizes, NPs can pass through the biological membranes and interact actively with cell organelles and cellular structures such as proteins, DNA, and RNA [1], and hence, they can be used as delivery vehicles for drugs to improve their circulation time and bioavailability [6].

Cancer is one of the major causes of death worldwide. Patients, when treated with traditional chemotherapy, suffer from various side effects, as small molecule drugs are non-selective and affect normal cells or tissues along with cancerous cells [7]. Within cancerous cells, the small size of NPs can enhance the concentration of the drugs inside the tumor by preferential accumulation due to the large pore size of neo-vasculatures and defective lymphatic system, which is defined as an enhanced penetration and retention effect [8]. 

Different types of NPs that are frequently used in medicine include metallic nanoparticles, dendrimers, liposomes, carbon nanotubes, and nanocrystals [9]. Among these, magnetic iron oxide NPs, particularly maghemite (ɤ-Fe_2_O_3_) and magnetite (Fe_3_O_4_) with a size of less than 20 nm, exhibit superparamagnetic behavior. These structural properties make the NPs ideal for several biomedical applications, including magnetic resonance imaging (MRI) [10], magnetically guided drug delivery [11], magnetic hyperthermia [12], and biosensors [13].

On the other hand, gold NPs are the most widely used and studied due to their easy preparation, high stability, well-established surface functionalization chemistry, and unique optical properties. Furthermore, gold NPs exhibit localized surface plasmon resonance providing efficient absorption and scattering of light at specific wavelengths. These wavelengths can be tuned according to their shapes and sizes. This property makes gold NPs an ideal choice as calorimetric sensors and nanoprobes for surface-enhanced Raman spectroscopy (SERS) and labels for imaging or photothermal agents for cancer therapy. Gold NPs can be easily functionalized using thiols and amines, while iron-oxide NPs can be functionalized using catechols, silanes, diol carboxylic groups, or amines [14,15]. 

In the last few years, new composite nanostructures have attracted considerable attention, particularly those based on bimodal metals, such as gold (Au) and iron (Fe_3_O_4_). More specifically, since Au possesses excellent properties as mentioned earlier, the construction of Au–iron oxide-based nanostructures would be extremely valuable providing complementary modalities for accurate imaging or diagnosis or offer an additional therapeutic avenue for enhanced cancer treatment, thus overcoming the limitation of individual treatment approaches [16]. Moreover, these nanohybrids effectively incorporate enhanced properties such as magnetic response, light absorption, and thermal effect in one nanoplatform [17]. Au NP catalysts were used in applications such as carbon monoxide oxidation, the reduction of aromatic nitro compounds etc. Although small volumes of Au NPs are required for catalysis, large amounts are still needed to carry out processes at industrial scale, which is uneconomical. One way to achieve low-cost gold-based catalysts is to create a gold hybrid. Larson and co-workers demonstrated superior catalytic properties for iron–gold NPs when compared to gold nanoparticles themselves [18]. 

Iron–gold-based NPs facilitate the attachment of different functional groups, allowing the conjugation of different drugs and can possess multiple functionalities in one single nanostructure coming from two different domains [19,20]. Indeed, the combination of plasmonic and magnetic properties is extremely appealing for biomedical applications, including hyperthermia [16], protein biomarkers detection [21], targeted delivery of DNA, siRNA [22] drug solubility improvement [7], tumor imaging, gene expression product detection, and targeted drug delivery [23]. These encouraging features together with the ease of surface modification make iron oxide–gold nanohybrids a robust platform for manifold biomedical applications.

Some excellent reviews have been published on the use of iron oxide–gold-based NPs in the field of nanomedicine [24]. However, we have reviewed important findings focusing on advances of iron oxide–gold-based structures in recent years. Herein, we discuss the general properties of nanoparticles, iron oxide, and its surface functionalization with inorganic and organic compounds, synthesis, and structures of hybrid iron–gold NPs, as well as a brief insight into biomedical applications of iron–gold NPs and their toxicity.

## 2. Properties of Nanoparticles

The novel properties and versatile features of NPs make them a preferred choice for a variety of applications in different fields, such as nanofabrication, surface engineering, and tribology [25]. These unique features of NPs are attributed to their notable size-dependent characteristics which are directly linked to their increased surface area. The size-to-surface ratios of NPs changes the atomic density of particles and give them their distinct physiochemical or mechanical characteristics from the bulk material. NPs with superior mechanical properties relative to the micro or bulk particles improve the strength and quality of processes such as microfabrication, which affect the tribological features of lubricants [26] and strengthen composite coatings [27]. An in-depth knowledge about their mechanical and physicochemical properties, such as elastic modulus or hardness, surface charge, adhesion or friction, colloidal stability, effects of size, and ligand chemistry, is usually needed for many practical applications. 

### 2.1. Hardness or Elastic Modulus of Nanoparticles

Hardness and elastic modulus are among the basic mechanical properties of NPs, and generally, a detailed characterization should be performed. The elastic modulus of NPs can be measured by compressing or bending the NPs by AFM, and in the case of spherical polymer nanoparticles, a uniform size-dependent, mechanical property is not observed thus far. Ramos et al. have reported that the elastic modulus and hardness of six-fold symmetry gold nanoparticles are greater than those of the bulk phase due to the production of stacked faults and dislocation in crystallographic direction [28]. 

### 2.2. Adhesion or Frictional Effects in Nanoparticles

The adhesive and frictional forces in NPs play a key role in determining their colloidal stability, lubrication, nanofabrication, nanodevice design, and drug delivery. Interactions of NPs with polished surfaces, in addition to good control on their mechanical properties, are crucial for improving the surface quality and enhancing material removal [29]. The adhesion of gold NPs (AuNPs), which are prepared on silicon substrates with a diameter of 25 nm, has been reported to decrease in the presence of hydrophobic interfaces [30]. In another study on spherical AuNPs, Maharaj et al. have reported that friction and wear of AuNPs decrease when water is added. AFM can be employed to observe interactive forces among NPs, the AFM tip probe, and the substrate, including the Van der Waals (VDW), electrostatic, capillary, frictional, and repulsive contact forces [31]. Moreover, several types of motions such as sliding, rotation, and rolling were also observed to be important when dealing with friction at nanoscale [32].

### 2.3. Effect of Size

The NP size crucially affects the type of particle location inside the body [33,34]. For instance, in a study by Carlos et al., 10 nm and 30 nm gold NPs accumulated more in liver and kidney as compared to 60 nm ones, which have been found more in spleen post-injection in Wistar rats [35]. If the size of NPs is large, it poses a physical difficulty to cross the lipid membrane of the cell. The size of NPs as well as the density of the receptor are key parameters in determining the particle uptake through receptor-mediated endocytosis [36].

### 2.4. Effect of Surface Charge or pKa

The increase in the surface charge or ionization leads to the increase in the uptake driving force of NPs, thereby permitting them to translocate the cell membrane easier. It serves as opposition to the influence of increased size. The size of the NP can be easily manipulated when compared to increasing the surface charge or ionization [37,38].

### 2.5. Effect of Ligand Chemistry

Ligand chemistry is another important factor when considering the translocation of NPs through the membrane. By changing the hydrophobic nature of NPs to a hydrophilic nature, the passage of NPs through the membrane is enhanced rather than the NPs being embedded inside it or attached to the surface [37]. The processing of AuNPs with hydrophobic ligands has been proposed to enhance targeted delivery and facilitate various diagnostic and therapeutic applications [39]. Overall, the enhancement of the hydrophobic nature of NPs aids in trapping the NPs inside the membrane due to the enhancement in the enthalpic reactions between the membrane and ligand. When NPs are placed inside the lipid membrane, those comprising high hydrophobic ligands exhibit high free energy gains compared to NPs comprising less hydrophobic ligands, helping NPs to be entrapped inside membranes [37,38].

### 2.6. Plasmonic Nanoparticles

Interactions of metallic NPs with light acquire a collection of free electrons called plasmons, further producing the surface plasmon resonance (SPR). The localized SPR phenomena generated by noble metal NPs such as gold are popular due to their resonance frequencies falling in both visible and near-infrared regions [40,41]. The LSPR phenomenon is studied and exploited for biomedical applications such as photo-induced hyperthermia and photo-induced bioimaging [14,41,42]. 

The size, morphology, and composition of noble metal NPs needs to be precisely controlled, keeping in mind the permittivity of the surrounding medium to develop a robust application-oriented LSPR peak suitable for biomedical applications [40].

Properties of plasmonic NPs such as optical scattering as well as mechanical and photothermal features depend on their surface properties, heat capacity, optical pulse length, and clusterization state [43].

### 2.7. Magnetic Nanoparticles

By exploiting the high magnetization property with a functionally designed surface, Fe_3_O_4_ NPs can be successfully attached to the target cell or tissue, in a selective manner; hence, magnetic NPs can be used in different medical fields from diagnosis to treatment. To this end, the particle surface can be functionalized so that NPs appropriately target a ligand and also carry a drug load to the desired target via external or internal stimuli [44]. Various strategies have been adopted for surface functionalization with the use of different coating agents and various biomolecules that ensure the ability of magnetic NPs to reach the desired target. A summary of critical properties of iron–gold NPs and their implications is compiled in Table 1.

## 3. Iron Oxide Nanoparticle Functionalization

For numerous biomedical applications, it is necessary to modify the NPs’ surface with a suitable shell. Modification is crucial due to the following reasons:

1. Due to their nanometer size, surface modification is required in order to reduce the surface energy.
2. Ligands bind inefficiently, and drug delivery fails if the surface of Fe_3_O_4_ NPs is not functionalized.
3. Uncoated Fe_3_O_4_ NPs can form free radicals.
4. To use Fe_3_O_4_ for biological applications that are inside the cells and proteins, it is appropriate to bind them with a suitable ligand for selective targeting [47].

**Figure 1:**
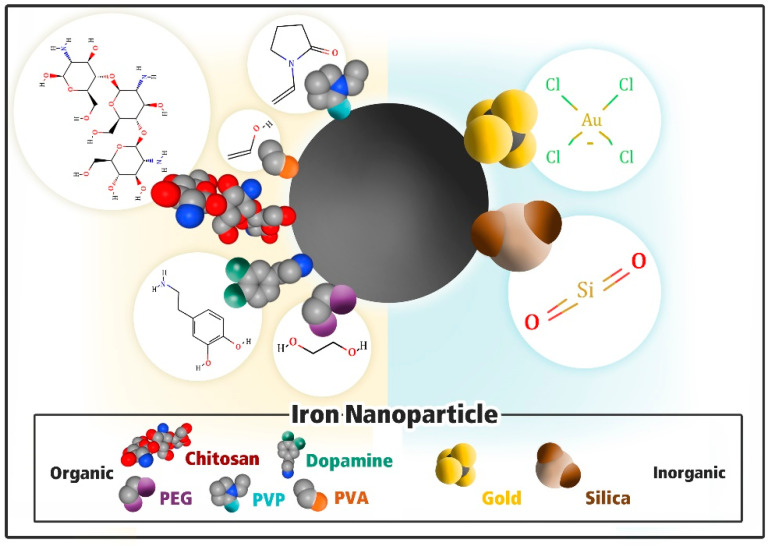
An iron oxide nanoparticle with different hydrophilic ligand molecules. The schematic representation of a surface modification of iron oxide nanoparticles. Left to right: organic to inorganic polyethylene glycol (PEG), polydopamine (PDA), chitosan, polyvinyl alcohol (PVA), polyvinyl pyrrolidone (PVP), gold, and silica. Molecule structures were taken from the pubchem.ncbi.nlm.gov and displayed as surfaces and modeled from their chemical structure with a space-filling model.

When the surface of Fe_3_O_4_ NPs is modified using organic or inorganic materials, nontoxic and biocompatible NPs are formed, which can be used for biomedical applications [48,49]. Inorganic materials such as gold and silica can be used to coat Fe_3_O_4_ NPs. These inorganic materials enhance the binding ability of the NPs to biomolecules and render increased stability to the iron–gold NPs surface. In addition, when Fe_3_O_4_ NPs are coated with organic materials, the agglomeration of these Fe_3_O_4_ NPs in the solution is prevented [47]. Figure 1 provides the schematic representation of the commonly used molecules to functionalize the iron nanoparticles.

### 3.1. Modification with Inorganic Material

#### 3.1.1. Gold (Au)

Gold enhances the functionality and stability of magnetic NPs in aqueous solutions. In addition, it exhibits optical properties due to Au’s surface plasmonic resonance (SPR) [50]. The process involves the reduction of an Au precursor in the presence of Fe_3_O_4_ nanoparticles. Iron–gold NPs can be synthesized by the reduction of HAuCl_4_. However, this Au coating process is difficult to do at room temperature due to the incompatibility of the underlying chemistry [51]. Alternatively, an iron–gold shell can be constructed at 180–190 °C using 1,2-hexadecanediol as a reducing agent. At high temperatures, surfactants are adsorbed on the iron–gold NPs’ surface [52]. Generally, TEM is employed to determine the thickness of nanoparticles [53].

#### 3.1.2. Silica (SiO_2_)

In a colloid system, SiO_2_ is used as an inorganic coating for the NPs surface. A silica shell over the NP surface renders protection against toxicity, enhances chemical stability, and prevents the aggregation of iron–gold NPs in the liquid phase [54]. Stabilization induced by silica coatings on the surface of Fe_3_O_4_ NPs occurs in two different ways: the first one is by protecting the dipole interaction with the shell of silica. Secondly, by enhancing the Coulomb repulsion of Fe_3_O_4_ by silica [55]. 

### 3.2. Modification with Organic Materials

#### 3.2.1. Polydopamine

Dopamine is a biomaterial derived from dihydroxy-L-phenylalanine (DOPA), and it belongs to the class of catecholamines, which plays a significant role in achieving adhesions to muscles. It can interact with multiple substrates via covalent and non-covalent bonds [56]. Polydopamine (PDA) is a versatile polymerization product of dopamine monomers that can attach to metallic surfaces [56]. An organic solvent is not required in the synthesis of PDA due to its strong adhesive properties. Diverse particle sizes can be achieved by the surface modification of PDA by varying the reaction conditions, including the dopamine concentration, pH, reaction time, temperature, and oxidants [57]. PDA modifications enhance the drug-loading ability of the NPs, making them effective drug carriers [56].

#### 3.2.2. Poly Vinyl Pyrrolidone (PVP)

Poly vinyl pyrrolidone is a water-soluble polymer, and because of its distinct properties such as high chemical and thermal resistance, unique wetting, binding, and film-forming properties, it is widely used in various biomedical applications [58]. It exhibits a neutral charge, as well as biocompatibility and good aqueous solubility. A PVP coating is strengthened by covalent bonds, and it is known to enhance the stability of superparamagnetic iron oxide NPs (SPIONS) [59]. PVP-coated iron NPs can be efficiently used as MRI contrast agents [60,61]. 

#### 3.2.3. Chitosan

Chitosan is a biopolymer with diverse physical and chemical properties including but not limited to possessing high charge density at pH < 6.5, amenability for chemical modification, and bacteriostatic in nature [62], rendering it with novel characteristics and functions; thus, it can be extensively used in biomedical applications. This biopolymer is environmentally friendly, biocompatible, non-antigenic and nontoxic [63]. Therefore, it is beneficial to encapsulate Fe_3_O_4_ nanoparticles within chitosan. The amino and hydroxyl groups on chitosan form complexes with the Fe_3_O_4_ surface, providing stability and biocompatibility. For therapeutic drug delivery and MRI, the positive charge of its amino group interacts with the negative charge of the nucleic acid, making it biocompatible [64].

#### 3.2.4. Polyethylene Glycol (PEG)

Polyethylene glycol is a biocompatible biopolymer. A study by Nathan et al. demonstrated that PEG enhances the dispersion of iron oxide NPs and the stability of NP solution [65]. In addition, PEG can be linked to different biomolecules by serving as a spacer [66]. PEGylated superparamagnetic iron oxide NPs (SPIONS) demonstrated highest relaxation times, a requirement for better MRI imaging, and were resistant against aggregation [67].

#### 3.2.5. Polyvinyl Alcohol (PVA)

PVA is a biopolymer with widespread applications in biomedicine. When an iron–gold NPs’ surface is coated with PVA, PVA reduces agglomeration, and the NPs show good monodispersity [68]. PVA provides high biocompatibility, resistance to protein adsorption, and cell adhesion [69]. Therefore, for the coating of SPIONs, PVA is a good biocompatible material with excellent water solubility.

## 4. Iron–Gold Bifunctional Nanoparticles

Owing to advances in synthetic methodologies, bifunctional iron–gold NPs with various shapes, sizes, chemical, and physical properties are being extensively tested in biomedical applications including targeted drug delivery [23], biosensing [70], photothermal therapy [71], and different immunoassays [72]. Compared to other NPs, bifunctional iron–gold nanoparticles exhibit significant benefits due to their small size, large surface-to-volume ratio, optical characteristics, and high magnetic properties [73]. The use of these NPs in the aforementioned applications depends on their properties, i.e., slow oxidation, increased magnetic susceptibility, low toxicity, and high saturation magnetization. 

### 4.1. Structure of Iron–Gold Nanohybrids

**Figure 2:**
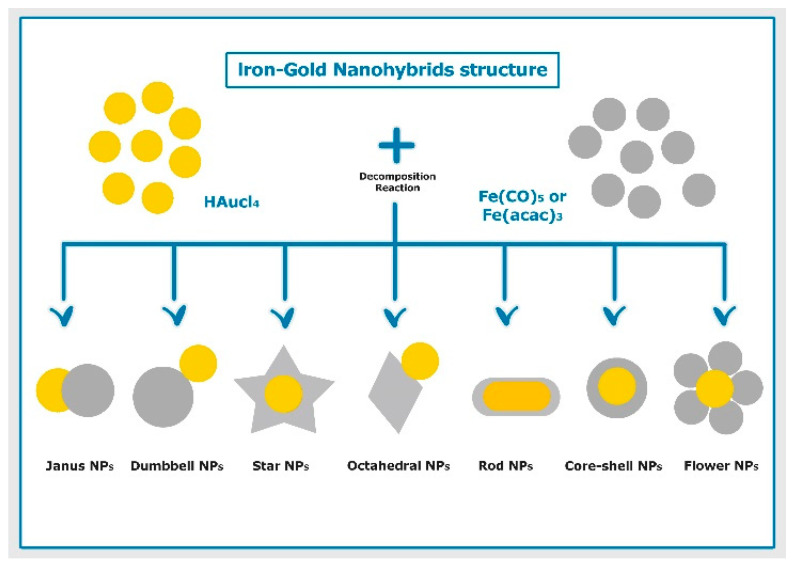
Schematic illustration of different structures of iron–gold hybrid nanoparticles prepared using thermal decomposition method.

Depending on their use, iron–gold NPs can be designed in various structures. The appropriate particle structure can be obtained by different factors, including particle shape, size, and chemical composition. The most desired morphologies of the iron–gold NPs include core–shell, dumbbell-shaped, Janus-shaped, flower-shaped, star-shaped, octahedral-shaped, and rod-shaped, as depicted in Figure 2. The synthesis of these morphologies, shape, and final performance depends on their junctional mode and interfacial morphology of the heterodimer structures [74]. 

Randomly decorated NPs are formed by the random joining of components during the seed-mediated synthesis of the heterodimers. Large building blocks are the hosts of the guest particles, and the used components can form different shapes, including irregular non-geometric, spherical, or irregular [75]. Different morphologies result in differences in the biocompatibility and magnetic properties of the NPs and lead to increased biocompatibility, magnetization, and specific absorption rates [76].

#### 4.1.1. Core–Shell Shape 

A core–shell structure is composed of an inner core coated with one or more layers (shells) of different materials. The core–shell structure has an advantage over other morphologies due to its high stability and tenability [77] and possesses a small size and large surface area [78]. 

Core–shell nano-heterostructures based on iron and gold can be prepared via a seed-assisted method by the incorporation of a reducing agent and slowing down the heating process [79].

Iron–gold core hollow shell nanoparticles demonstrate potential for use as MRI agents [80]. The selection of core and shell depends on the kind of application. For instance, for drug delivery purposes, the core can be iron and the shell can be silica/polymer; whereas for separable catalysis, the shell would be a metal nanocluster [81]. For designing a cancer nanotherapeutic that can perform chemotherapy/photothermal effect and multimodal cancer imaging, the magnetic core can be used for imaging, and a secondary coating layer with a porous silica layer can be used for encapsulating a chemotherapeutic agent. Lastly, a second shell coating with gold nanorods can give photothermal effects. Furthermore, the core–shell structure can retain the size, structure, and catalytic activity of gold NPs under thermal and mechanical stress when covered by metal oxides [82].

The incompatible surface energies of gold and iron make it difficult to construct core–shell structures. During preparation, parts of the iron oxide core are often exposed due to incomplete coating, which further causes heterogenous biofunctionalization and dysfunctional optical applications. One strategy to overcome this problem is to include an intermediate layer between iron and gold in the core–shell structure [83]. 

#### 4.1.2. Dumbbell Shape 

**Figure 3:**
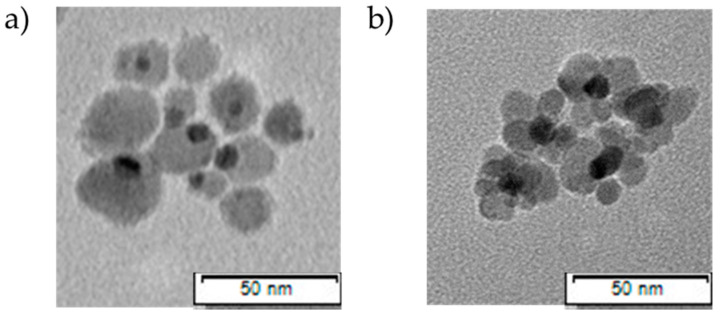
Representative TEM micrographs illustrating iron–gold nanostructures: (**a**) nanodumbbells, (**b**) nanoflowers taken from Kayser et al. (unpublished).

Dumbbell-shaped nanoparticles were reported first in 2005 [84]. In this morphology, local changes in the electronic structure occurs along the point of attachment between iron and gold, resulting in the alterations in the magnetic, optical, and catalytic properties of NPs [85]. A TEM micrograph of dumbbell-shaped iron–gold NPs has been recorded as part of a study by our group, as seen in Figure 3a (Kayser et al. unpublished). Dumbbell-shaped Au-Fe_3_O_4_ systems have numerous advantages: (1) They can be made directly without any pretreatment [84]. (2) They have both optically active plasmonic (Au) unit and magnetic (Fe_3_O_4_) properties, which makes them ideal for both magnetic and optical detection. (3) The presence of both particles on the surface makes it favorable for target-specific delivery and imaging functions. (4) They support drug release and cell targeting, as the small particle size provides an advantage to accommodate therapeutic molecules, proteins, antibodies, and a few strands of DNA [86]. Studies on the dumbbell heterodimers have demonstrated that the symmetry and free surface areas of dumbbell heterodimers are greater than those of randomly decorated ones, making them desirable for use in photo- and bio-catalysis applications [87]. The synthesis of the iron–gold heterodimers by the seed-mediated technique of non-spherical magnetic particles can affect their plasmonic properties during the interfacial morphology [74].

#### 4.1.3. Janus Shape 

Janus-shaped NPs are heteroparticles with two chemically different domains. These NPs exhibit unique structures with different surface characteristics and similar shapes and dimensions. Their morphology and composition render multimodal and versatile capabilities for use as drug delivery carriers and contrast agents in future medicine [88]. Owing to their resemblance to natural biomolecules, Janus-shaped NPs are suitable for use in biomedical applications, and they exhibit improved biocompatibility as well as magnetic and plasmonic properties [89]. Moreover, Janus NPs can accommodate drugs with different solubilities without influencing each other in their two distinct domains and release drugs independently by different stimuli. For instance, in a study by Zhang et al., poly(3-caprolactone)-gold nanocage/ferric hydroxide-poly (acrylic acid) Janus-shaped NPs were developed and loaded with two anticancer drugs, i.e., a hydrophilic doxorubicin and a hydrophobic docetaxel in two domains. These Janus NPs demonstrated independent control release of docetaxel and doxorubicin as a function of pH and NIR, respectively [90]. 

Furthermore, the unique anisotropic surface properties of Janus-shaped NPs allow them to house different functionalities in one nanoplatform. For example, in a study by Qi Zhang et al., the Au component of Janus NPs were functionalized with folic acid and poly (ethylene glycol) to obtain cancer targeting and high contrast for X-ray computed tomography. Concurrently, the Fe_3_O_4_ component acted as a delivery vehicle for doxorubicin, a magnetic resonance imaging contrast agent and a photothermal therapy agent [91]. Hence, both the components in Janus NPs can be functionalized and together provide an excellent nanoplatform with multiple functionalities due to these unique properties.

#### 4.1.4. Flower-Shape

Flower-shaped NPs are comprised of three to five petals of iron oxide and a core Au, as seen in Figure 3b (Kayser et al. unpublished). There are only few studies so far on flower-shaped iron–gold NPs [20,92]. These flower-shaped nanoparticles have been successfully used as efficient matrices for laser desorption and ionization mass spectrometry (LDI-MS). They can be utilized to target cancer cells and capture ATP molecules in the detection and analysis of metabolites from cancer cells and molecular imaging [20,92].

#### 4.1.5. Star Shape

Star-shaped heterodimers are synthesized from the Janus particles and exhibit slight morphological changes. Moreover, these NPs exhibit improved plasmonic and magnetic properties in comparison with those of randomly decorated and spherical structures [14]. As such, these NPs can be effectively used as contrast agents for different imaging techniques, such as X-ray computed tomography, MRI, optical microscopy, and photoacoustic imaging [14]. 

Furthermore, the spiky morphology of the Au imparts an enhanced electric field to the NP due to focalization of the plasmonic electromagnetic field at the sharp tips and edges. This local amplification effect allows highly sensitive magnetic detection of target molecules representing an interesting application for SERS detection using Fe_3_O_4_@Au nanostars [93]. Nguyen and co-workers reported the magnetically-assisted surface-enhanced Raman spectroscopy (SERS) performance of Fe3O4@Au nanostars for detection of traces of thiram, a popular pesticide, in water. Fe3O4@Au nanostars were able to detect thiram at solute concentrations as low as 10^−8^ M [94].

#### 4.1.6. Octahedral Shape

Octahedral-shaped NPs have bulk magnetic properties, and because of their shape and high magnetization, they have superior in vitro and in vivo T2 relaxivity and can be used as contrast agents in MRI. These NPs are comprised of 20–30 nm magnetite octahedral nanocrystals, and they are grown on Au NPs by modified-wet chemical synthesis. These NPs exhibit two functional surfaces that are suitable for theranostic applications, including as the delivery of drugs in cancer treatment and dyes in intravital microscopy [95]. Octahedral NPs have demonstrated superior characteristics such as high relaxometric efficiency compared to commercial T_2_ contrast agents and excellent heat dissipating capacity in vitro [96].

#### 4.1.7. Rod Shape 

The polymer-coated gold nanorods upon mixing with iron salts and base resulted in iron-coated gold nanorods. These nanorods exhibited milder response under the application of an externally applied magnetic field (AMF) [97]. This is because magnetic manipulation is a lengthy process, and moreover, due to the thinness of the magnetic coating compared to the high mass of the core gold, these iron oxide-coated gold nanorods demonstrated poor response to AMF. However, nanorods formed by electrostatic interactions of pre-existent iron NPs and gold nanorods were more responsive to AMF. Nevertheless, they demonstrated problems in the reproducibility and uniformity of iron oxide coating [97]. In another study by Brian et al., premade iron NPs were stabilized by hydrophobic ligands and allowed to aggregate with silicon-coated gold nanorods present in non-polar solvent. The iron–gold nanorods prepared by this method were highly responsive to AMF. When compared to other methods, the heteroaggregation approach is simple and efficient for preparing iron oxide–gold nanorods due to the adjustable polarity of the solvents [98]. 

A comprehensive list of the different structures and properties of iron–gold NPs is summarized in Table 2.

### 4.2. Particle Synthesis of Iron–Gold Nanohybrids

In the past decade, several synthetic methods for iron–gold NPs have been reported. Herein, we will discuss the thermal decomposition protocol, which is the most widely used approach to synthesize iron–gold NPs. Thermal decomposition has been further developed for the large-scale production of nanodimers, which is referred to as one-pot and two-pot synthesis methods. In two-pot synthesis, gold NPs are separately prepared and subsequently introduced into a mixture of 1-octadecane, oleic acid with oleylamine, which is used as the solvent. Iron salt is introduced at 150 °C and heated until 300 °C. In one-pot synthesis, a gold precursor salt, HAuCl_4_ is introduced at 120 °C, affording Cl^−^ ions that control the dimensions of the iron-oxide moieties attached to the gold seed. Iron salt is added to this solution at 150 °C, and temperature is subsequently raised to 300 °C to decompose Fe(CO)_5_, resulting in the formation of dimers with a narrow size distribution [84,100]. With the start of nucleation of Fe, the free electrons from Au compensate for the charge induced by the polarized plane at the interface. As a result, Au NPs become deficient and unsuitable for multi-nucleation, resulting in a dumbbell-shaped structure. Once the synthesis of these dumbbell-shaped structures is completed, the nanoparticles are exposed to air, and Fe is oxidized to either Fe_3_O_4_ or ɤ-Fe_2_O_3_. NPs are dissolved in hexane in the presence of oleylamine for long-term stability as a dispersion [101].

In 2008, Yin et al. [102] have adjusted the Au-Fe molar ratios by following the same method to synthesize nanohybrids in size ranges of 2.5–3.5 nm and 15–16 nm, followed by oxidization with CO. Wang et al. [103] have proposed slight modifications of this method by introducing absolute ethanol after heating the mixture at 300 °C for 20 min. 

Using Au seeds of 3 and 5 nm-sized particles, Pablo et al. synthesized iron oxide-gold NPs with a dimer-like morphology owing to a single iron oxide nucleation event. This method is different than the multi-nucleation event, where the incorporation of a reducing agent or decreasing the reaction temperature leads to the formation of core–shell structures. However, increasing the size of Au seed to 8 nm yields to a flower-like structure [79].

In one method, silane-poly (ethylene glycol) and thiol-poly (N-isopropyl acrylamide) was used, which binds to the iron oxide and the gold surfaces, respectively. Gold nano-stars are grown by the injection of the dumbbell seeds in pre-reduced DMF solution of HAuCL_4_ at room temperature [14].

As a variation of the above method, after growing the Fe_3_O_4_ on Au-seed particles, 1-octadecene is replaced by phenyl ether, which increases the reaction time from 45 min to 3 h; the increase in the reaction time allows for complete crystallization. The NPs produced by this method exhibit strongly improved crystallinity and highly faceted growth mode based on an octahedral motif. In addition, the Janus NPs produced by this method are stable and non-toxic, and these NPs exhibit improved bulk-like magnetic properties [96].

As another variation, snowman-shaped gold–iron oxide nanohybrid dimers were obtained by the thermal decomposition of iron (III) oleate on pre-synthesized gold NPs. The gold NPs were synthesized in chloroform via reducing HAuCl_4_ with tert-butylamine borane in the presence of oleylamine. The size of the gold NPs was adjusted to a mean value of 6 nm by tuning the HAuCl_4_/oleylamine ratio. The morphology of the Au−Fe_3_O_4_ was found to be dependent on the iron (III) oleate concentration and size of the pre-synthesized AuNPs [104].

### 4.3. Biomedical Applications of Iron–Gold Nanohybrids

The combination of plasmonic and magnetic components has opened new venues for new applications. Selected examples of nanohybrids used in specific applications including drug delivery, hyperthermia, bioimaging, and biosensing are reviewed in the following section.

#### 4.3.1. Iron–Gold Nanohybrids as Delivery Carriers

Nanohybrids combining magnetic and gold nanoparticles facilitate the selective release of drugs [105] which are successfully employed for gene [106] and drug delivery applications [23,107,108,109,110]. For instance, Reza et al. has reported a nanohybrid of iron oxide and gold NPs with a polymer such as PVA and cytotoxic drug such as docetaxel. In this study, a magnetic core is formed, which was coated by a silica layer, and docetaxel was subsequently encapsulated inside the PVA gel layer formed outside the silica-coated iron NPs. Finally, gold nanoparticles were attached under green light irradiation, leading to a functional anticancer multi-stimuli nanohybrid system. This nanohybrid system exhibited temperature-triggered drug release in vitro in cancerous and normal cells. Moreover, an increase amount of docetaxel was released in cancerous cells due to acidic pH, indicating the efficiency of nanohybrid for selective delivery. Overall, the tumor inhibition was achieved by a combination of magnetically targeted delivery by iron NPs, pH and temperature-selective docetaxel release by PVA component, photothermal effect of gold NPs, and anti-tumor effects of docetaxel. This study reflects the successful report of iron–gold nanohybrids as a single system for multifunctional uses in cancer therapy [105]. In another example, Yun Quan-Li et al. have covalently attached a cytotoxic agent such as methotrexate to iron–gold alloy NPs and reported that with the increase in the applied magnetic field, the release of methotrexate increased in an incremental manner [111].

Singh et al. have reported the self-assembly of iron–gold core–shell nanoparticles with N-Nitrosothioproline (NHTP), which is a nontoxic biological molecule that can release nitric oxide (NO), which is a cytotoxic agent on cleavage. The differential release of NO is observed from nanohybrids under different wavelengths of light, i.e., red, visible, white, and different time points, demonstrating the controlled-release effect of NO from nanohybrids. Furthermore, these nanohybrids are successfully employed for killing cancer cells (HeLa) in vitro [112]. 

Wi Shi et al. have developed iron–gold dumbbell NPs coated with polyethylenimine (PEI), a transfection agent, and then, they subsequently used these NPs as gene carriers to deliver green fluorescent protein in HEK 293T cells in vitro [106]. 

Lastly, dumbbell-like nanoparticles containing two different chemical surfaces, as well as being magnetically and optically activated, are particularly suitable for synchronized cell targeting and pH-sensitive drug delivery [113]. Overall, these studies demonstrate that gold and iron based nanohybrids can be used for the controlled release of drugs and selective drug delivery.

#### 4.3.2. Iron–Gold Nanohybrids for Hyperthermia Applications 

Hyperthermia is an experimental cancer therapy, where the temperature is raised either locally or regionally in the range of 40–46 °C or for the whole body to destroy cancer cells and inhibit the growth of tumors [114,115]. Hyperthermia primarily aims to selectively kill malignant cells without causing any harm to normal cells [116]. There has been a great deal of research aiming to precisely position heat-delivery applicators such as catheters, microwave, and ultrasound applicators near or around the appropriate region, or administering nanoparticles and energy is focused on the tumor to raise its temperature [115].

Iron–gold NPs are the most extensively investigated NPs for hyperthermia-induced cancer therapy. Noninvasive alternating magnetic field (AMF) causes fluctuations in the magnetic moment of iron-oxide NPs, leading to the conversion of magnetic energy to thermal energy (magnetic hyperthermia) [117,118]. On the other hand, by the application of an infrared laser, AuNPs can convert light energy into heat energy, leading to the localized thermal ablation of tumor cells (photothermia) [15,119]. The size and shape of iron and gold nanoparticles play a crucial role on their properties. In fact, for enhancing the heat-generating capacity of magnetic nanoparticles, appropriate magnetization, control of size, fine tuning of shape, and anisotropy is essential [120]. For example, magnetic cubic NPs demonstrate superior heating capacities in comparison with those of spherical magnetic NPs [121]. Similarly, gold nano shells produce more heat compared to gold nanorods, even though both exhibit the same densities [122]. Furthermore, the addition of AuNPs has been reported to increase the magnetically induced heat generated by iron-oxide nanoparticles [123].

Espinosa et al. have fabricated a multifunctional nanohybrid composed up of a multi-iron core and gold branched shell. Under the application of a magnetic field and laser irradiation, the resulting nanohybrids with varying gold branches exhibit a higher heat dissipation than that of either of them in vitro after dispersing in water. In vivo studies following intra-tumoral injection in mice showed that the iron oxide–gold nanohybrid caused a 20 °C increase in the temperature within two minutes of the applied magnetic field and NIR exposure, and the heating performance lasts for three additional days, reflecting the advantage of incorporating dual hyperthermia modalities for cancer therapy [124]. In a similar approach, Janus nanoparticles comprising an iron oxide nanosphere and a gold nanostar exhibited a synergistic tumor ablation under the application of magnetic field and photothermal treatment compared to stand-alone procedures. Moreover, the magnetic-guided accumulation of Janus nanoparticles into tumors is observed after the intravenous administration of these multifunctional nanoparticles, leading to on-site delivery [125]. Photodynamic therapy (PDT) is another type of light-catalyzed process that uses a photosensitizer (PS), which is a molecule that generates high-energy cytotoxic reactive oxygen species to destroy cancer cells [126,127]. Nevertheless, the use of PSs is mainly hindered due to the presence of their main absorption band lying in the ultraviolet-visible (UV-vis) region, wherein light penetration into the tissue is shallow. In addition, PSs exhibit a lack of specificity toward tumors [128]. Hence, nanocarriers have been designed to improve their NIR absorption for enhanced light penetration and target specificity [129,130]. Methodologies that blend PDT with additional treatments have demonstrated synergistic effects in cancer therapy [131,132]. For instance, Saheel Bhana et al. developed a new type of nanohybrid comprising of an iron-oxide core and gold nano-popcorns with strong NIR absorption. This absorption has been achieved by coating the nano-popcorns with NIR-absorbing photosensitizer silicon, such as 2,3-naphthalocyannie dihydroxide and stabilization with PEG. This nanohybrid demonstrated superior photothermal and photodynamic properties with the aid of magnetic field-guided drug delivery in comparison with the combination treatment without using a magnetic field, and the two treatments alone indicating the significance of multifunctional composites based on iron and gold in hyperthermia [133]. However, hyperthermia is under study in clinical trials and is not widely available [134].

#### 4.3.3. Iron–Gold Nanohybrids for Bioimaging

Multifunctional NPs have made possible theranostics for simultaneous imaging and therapy, as well as multi-modal imaging combining two or more imaging modalities. Dual surface-functionalized Janus nanocomposites of polystyrene/Fe_3_O_4_@SiO_2_ have been used for targeting tumor cells and simultaneously releasing drugs under an acidic intracellular environment [95]. Oxaliplatin–Au–Fe_3_O_4_–herceptin is a promising multifunctional platform for the simultaneous magnetic traceable and human epithelial growth factor receptor 2 (HER2) targeted chemotherapy for gastric cancer. The nanocomposite has probes for mass spectroscopy and nuclear magnetic resonance imaging as well as a drug-release assay that releases oxaliplatin (a breast cancer drug) in an acidic medium. The pH-controlled drug release lowers the dosage and period of administration for patients, thus reducing side effects and build-up of drug resistance in patients [135].

#### 4.3.4. Iron–Gold Nanohybrids as Biosensors

Hybrid NPs have also been used in nanostructure-assisted laser desorption ionization mass spectrometry (nano-LDI). With advanced mass spectrometry instrumentation, NPs have been used as a matrix for ionization and analysis of biomolecules. This technique is known as matrix-assisted laser desorption/ionization mass spectrometry (MALDI-MS). The use of NPs in MS compared with the use of organic matrices makes sample preparation easier, with higher salt tolerance, absence of self-ionization, and more rapid data acquisition. NPs have been used in a dual “catch and detect” mode, serving as a capturing probe as well as an ionization matrix. Surface-functionalized with cationic legends, the AuNPs have been used to assess the cellular uptake of AuNPs in LDI-MS. This reported demonstrated that the AuNPs are quite stable in an intracellular environment to enable their use of a matrix. NPs can be used for targeting specific cells by conjugating them with aptamers. The cells can be targeted for rare protein capture, enrichment, drug delivery, and therapy [20].

## 5. Toxicity Assessment

With increasing studies and potential applications, it is imperative to assess the toxicity and biocompatibility of NPs. Due to their small sizes, NPs can cross cell membranes and perhaps the blood–brain barrier, thereby affecting intracellular structures and metabolisms. Gold NPs exhibit low toxicity due to their inert nature. However, the intake of iron nanoparticles leads to their accumulation in spleen, liver and lungs, and it was demonstrated that in some cases, they can cross the blood–brain barrier [136]. The toxicity of NPs is related to the production of ROS [137]. High ROS levels can disrupt DNA, alter protein structures and interactions, damage cells, modulate gene transcription, induce cell apoptosis and permeability, and change the cell morphology [138]. Specifically, these nanoparticles alter the blood coagulation systems, lead to cell lysis and inflammation, and reduce cell viability [139]. Gupta et al. have reported that the pullulan-coated iron NPs are nontoxic to human dermal fibroblasts comparing to the uncoated iron NPs [140]. Thus, the assessment of NPs is a major concern for the maintenance of the toxicity.

### 5.1. In Vitro Toxicity Assessment Methods

The in vitro assessment of NPs is feasible due to its cost-effective, rapid results, and ethical concerns. This type of toxicity assessment includes various assays, the details for which are explained below.

#### 5.1.1. Proliferation Assay

A proliferation assay is employed to measure cell metabolism by examining metabolically active cells. The most common tetrazolium salt used for in vitro toxicity of NPs in this method is 3-(4,5-dimethyl-thiazol-2-yl)-2,5-diphenyltetrazolium bromide (MTT) [141]. This assay involves the assessment of tetrazolium salt. The resulting efficacy depends on the reaction conditions. Thymidine incorporation aids in the assessment of cell proliferation, but it is not feasible due to its toxic effects and high costs [142]. Another assay known as cologenic assay is also employed to examine cell proliferation by enabling the visual count of exposed NPs [143]. A study demonstrated that cell viability decreased by 85% in 72 h after exposure to 100 µm nanoparticles [144]. In another study, Hu et al. reported that cytotoxicity was dose dependent when Fe_3_O_4_ nanoparticles were exposed on mouse macrophages for 1 and 4 days at 0.2 mg/mL [145].

#### 5.1.2. Apoptosis Assay

Apoptosis is programmed cell death process that occurs in physiological and pathological conditions [146]. It is the major detection marker during in vitro toxicity assessment of NPs. Iron–gold NPs are toxic at high concentrations and can influence apoptosis, although the surface modifications can greatly reduce their toxicity. These NPs can be characterized using UV-Vis spectroscopy, dynamic light scattering, and field emission scanning electron microscope [147]. Cellular damage following NP treatment can be identified by various methods, including endotoxin and lactate dehydrogenase (LDH) signaling and oxidative stress detection supply; these valuable techniques expose the biomarkers that induce cellular damage [148]. Agarose gel electrophoresis can aid in differentiating cells that have undergone apoptotic and necrosis [149]. The attachment of hapten moieties to annexin V permits the labeling of the apoptotic cell. The annexin V assay monitors apoptotic events, while the caspase assays are beneficial for the identification of cellular apoptosis signals. Other assays including fluorogenic and chromogenic caspase, immunoblotting, and immunofluorescence caspase can also be used in the identification of apoptosis [148].

#### 5.1.3. Necrosis Assay

Necrosis assessment enables the determination of cell viability by examining membrane integrity. When cells undergo necrosis following the uptake of iron–gold NPs, LDH is released into the surrounding extracellular environment, although its enzymatic activity is maintained [148]. The monitoring of the LDH enzymatic activity by using known concentrations of lactate and NAD via the use of enzymatic-linked immunosorbent assay (ELISA) or a UV-Vis spectrometer can indicate the rate of cell necrosis. During this process, membrane integrity can be examined using external dye binding with neutral red or trypan red [150]. These dyes cross the cell membrane and interact with lysosomes to induce surface changes, which in turn make the lysosomes fragile. Changes due to the NPs reduce the dye uptake and its binding, thereby permitting clear detection between viable and dead cells. Furthermore, cellular necrosis in cells can be identified by attaching hapten moieties to annexin V, enabling apoptotic cell labeling and secondary stains that indicate necrosis [148].

#### 5.1.4. Oxidative Stress Assay

Oxidation levels can be detected by the direct measurement of reactive oxygen species (ROS), when the structural alteration of NPs occurs, causing their uptake to lead to the ROS generation [151]. When clearance of these nanoparticles fails, it leads to oxidative stress, inflammation, and cell death. Moreover, uncleared NPs can further affect blood flow into organs, eventually leading to the rupture of blockages. Notably, the chemical composition and NP shapes greatly affect their interaction with the cells. Furthermore, ROS can be detected by using 2,2,6,6-tetramethylpiperidine (TEMP) following the electron paramagnetic resonance technique [152]. ROS can also be measured using fluorescent probes such as 5-(and -6)-carboxy-2,7-dichlorodihydrofluorescein diacetate (DCFDA) and dihydroethidium (DHE) [153]. Previously, gold NPs-induced DNA damage [154] and gold NPs causing autophagy with oxidative stress in human lung fibroblasts was shown [155]. 

### 5.2. In Vivo Toxicity Assessment Methods

This approach involves detection methods such as clearance, serum chemistry assessment, hematology, biodistribution, and histopathology. The clearance of NPs from body involves the estimation of excreted NPs at various periods after exposure [156]. Biodistribution measurements involve the examination of the NP localization routes in various tissues using radiolabels [157]. On the other hand, histopathology is employed to check the NP exposure in various tissues such as the eyes, lungs, liver, spleen, and kidneys [158]. In vivo studies have reported that the gold NPs induce apoptosis and acute inflammation in the liver [159]; however, similar studies involving other NPs are still lacking.

## 6. Conclusions and Future Directions

Hybrid nanoparticles have significant advantages in biomedicine because of their multifunctional capabilities. In particular, much progress has been made in the synthesis of iron–gold-based multifunctional hybrid NPs, and successful surface functionalization via different functional groups. Different structures of iron–gold-based hybrid nanoparticles have been evolved recently with unique properties corresponding to their structures.

Owing to their unique features, iron–gold hybrid nanoparticles have demonstrated to be ideal candidates as drug delivery vehicles in cancer therapy and several other biomedical applications. Their size and shape can be tuned by different synthetic methods and various surface modification approaches, making them suitable for a wide range of applications such as multimodal imaging, multimodal therapy, biosensing, and theranostics. Even though iron–gold NPs are becoming increasingly popular, considerable work still needs to be performed in terms of optimization of the synthetic routes to obtain better and robust nanohybrids for achieving enhanced targeted therapies. 

Moreover, thus far, most studies based on iron–gold nanoparticle hybrids are still in a proof-of-concept stage, highlighting a need to establish detailed preclinical studies. Despite possessing unique physicochemical properties suitable for biomedical applications, inorganic nanoparticles suffer from significant toxicity issues. Hence, to enter clinical trials for humans, it is empirical to evaluate the pharmacokinetics and biodistribution of these nanohybrids first in preclinical studies. A careful estimation of toxicity parameters such as lethal dose, maximum tolerable dose, and side effects must be performed. In addition, it is crucial to determine the minimum number of modalities required in a nanoplatform to get the desired efficacy for the effective treatment of cancer economically. Determining these parameters will certainly prompt the transition of novel iron–gold-based hybrid nanoparticles from bench to bedside.

## Figures and Tables

**Table 1:** A comprehensive list of properties and implications of nanoparticles.

Properties	Implication	References
Hardness or elastic module	Helps to better accomplish versatile goals and control action mechanisms.	[45]
Adhesion or frictional effects	Determines the colloidal stability, lubrication, nanofabrication, nanodevice design and drug delivery capabilities.	[29]
Nanoparticle size	Increasing the size enhances the physical difficulty to cross the lipid membrane of the cell and adversely affects the particle uptake through receptor-mediated endocytosis.	[36]
Surface charge or pKa	Increasing the surface charge/ionization increases the driving force of NPs and makes them translocate the cell membrane. It serves as opposition to the influence of enhanced size.	[37,38]
Ligand chemistry	AuNPs processing with hydrophobic ligands enhances targeted delivery and facilitates various diagnostic and therapeutic applications. The hydrophobic nature of the particle helps in trapping it inside the membrane by enhancing enthalpic reactions between the membrane and the ligand. NPs with high hydrophobic ligands possess high free energy gains as compared to the NPs with less hydrophobic ligands when placed inside lipid membranes and would help entrap the NPs inside the membrane.	[37,38]
Plasmonic	Plasmonic properties of noble metallic NPs are exploited for biomedical applications such as hyperthermia and bioimaging.	[14,41,42,46]
Magnetic	High magnetic properties with functionally designed surface of iron oxide NPs can be exploited in hyperthermia and image-guided delivery.	[12,16]

**Table 2:** Different structures of gold iron oxide nanoparticles, their properties and implications.

Structure	SIZE (nm)	Properties	Application	Implication	References
Flower	30–300 nm	Comprises three to five petals of iron oxide and a core Au	Targeted cancer, LDI-MS	Suitable for use as efficient matrices in LDI-MS. Use in biomedical applications, such as targeting cancer cells and capture of ATP molecules. Use in detection and analysis of metabolites from cancer cells and molecular imaging.	[20]
Star	≈70–150 nm	Improved plasmonic and magnetic properties	X-ray, MRI	Suitable as contrast agents for different imaging techniques, such as X-ray computed tomography, MRI, optical microscopy, SERS detection of target molecules, and photoacoustic imaging.	[14,93,94]
Dumbbell	≈10–60 nm	Optically active plasmonic and high magnetic properties	Photothermal, biocatalysis	High symmetry and large free surface areas make them desirable for use in the photo- and biocatalysis applications.	[87]
Core-shell	70 to 250 nm	High stability and tenability	Catalysis and cancer therapy	Colloidal stability in dispersion ensures a better shelf life.	[99]
Octahedral	25 nm	High magnetization High crystallinity, relaxometric efficiency	MRI	Superior in vitro and in vivo T2 relaxivity, suitable for use as contrast agents in MRI. Two functional surfaces make them suitable for theranostic applications.	[95,96]
Rod	8 nm	Enhanced plasmonic properties	Photothermal therapy	Robust iron oxide–gold nanorods can be prepared using the heteroaggregation approach	[97,98]
Janus	≈120 nm	Chemically different domains Anisotropic properties	Targeted cancer, simultaneous differential release of drugs	Suitable for multiple functionalities in one nanoplatform.	[90,91]

## Data Availability

Not applicable.

## Abbreviations

AFM	Atomic force microscopy
Au	Gold
Au−Fe_3_O_4_	Gold iron
DCFDA	5-(and -6)-Carboxy-2,7-dichlorodihydrofluorescein diacetate
DHE	Dihydroethidium
DMF	Dimethylformamide
DNA	Deoxyribonucleic acid
DOPA	Dihydroxy-L-phenylalanine
ELISA	Enzymatic-linked immunosorbent assay
EPR	Electron paramagnetic resonance
Fe(CO)_5_	Iron pentacarbonyl
Fe_3_O_4_	Iron magnetite
(ɤ-Fe_2_O_3_)	Maghemite
HAuCl_4_	Chloroauric acid
HEK 293T	Human embryonic kidney 293 cells
HER2	Human epithelial growth factor receptor 2
LDI-MS	Laser desorption and ionization mass spectrometry
LDH	Lactate dehydrogenase
MRI	Magnetic resonance imaging
MTT	3-(4,5-Dimethyl-thiazol-2-yl)-2,5-diphenyltetrazolium bromide
NAD	Nicotinamide-adenine dinucleotide
NHTP	Nitrosothioproline
NO	Nitric oxide
NPs	Nanoparticles
NIR	Near-infrared
PDA	Polydopamine
PDT	Photodynamic therapy
PEG	Polyethylene glycol
PEI	Polyethylenimine
PVA	Polyvinyl alcohol
PVP	Poly vinyl pyrrolidone
ROS	Reactive oxygen species
SERS	Surface-enhanced Raman spectroscopy
SiO_2_	Silica
SPIONs	Superparamagnetic iron oxide nanoparticle
SPR	Surface plasmon resonance
TEM	Transmission electron microscopy
TEMP	Tetramethylpiperidine
UV	Ultraviolet
VDW	Van der Waals
